# Comprehensive dataset on fluoride removal from aqueous solution by enhanced electrocoagulation process by persulfate salts

**DOI:** 10.1016/j.dib.2023.109492

**Published:** 2023-08-12

**Authors:** Hamid Reza Tashauoei, Mokhtar Mahdavi, Ali Fatehizadeh, Ensiyeh Taheri

**Affiliations:** a Department of Environmental Health Engineering, Faculty of Public Health and Biomedical Engineering, Tehran Medical Sciences, Islamic Azad University, Tehran, Iran; b Assistant Professor of Environmental Health Engineering Department, Saveh University of Medical Sciences, Saveh, Iran; c Social Determinants of Health Research Center, Saveh University of Medical Sciences, Saveh, Iran; d Department of Environmental Health Engineering, School of Health, Isfahan University of Medical Sciences, Isfahan, Iran; e Environment Research Center, Research Institute for Primordial Prevention of Non-Communicable Disease, Isfahan University of Medical Sciences, Isfahan, Iran

**Keywords:** Fluoride, Electrocoagulation, Oxidation process, Persulfate salts

## Abstract

Depending on the quantity and concentration, drinking water containing fluoride (F^–^) ions can have either favorable or unfavorable impacts on individuals and the environment. High levels of F^–^ (over 2 to 4 mg/L) can cause skeletal problems, dental fluorosis, and brain damage in children. Conventional F^–^ removal is often complex and thus causes an adverse effect on the environment and financial burdens. The use of persulfate salts to enhance the electrocoagulation process is one of the most recent advances in the removal of F^–^ from water. To investigate the efficacy of F^–^ removal, a laboratory-scale electrochemical batch reactor with iron and aluminum electrodes was employed with various persulfate doses, pH values, current intensities, and supporting electrolyte concentrations. It was observed that the performance of the enhanced electrocoagulation process by persulfate increased over time, and it worked well in a certain range of pH. Also, for the initial F^–^ concentration of 10 mg/L, increasing the supporting electrolyte concentration to 1.5 g/L improved fluoride removal efficiency from 80 to 91.2%, but higher concentrations (2.5 g/L) reduced efficiency to 71%. The most effective removal of F^–^ was found to occur at a persulfate dose of 0.2 mg/L. At this dose, F^–^ removal efficiency exceeded 92% for all studied F^–^ concentrations. Overall, electrocoagulation using persulfate salts proved more efficient than electrocoagulation alone at removing fluoride from water sources.

**Specifications Table**

**Table:** 

Subject	Environmental Science - Chemistry
Specific subject area	Fluoride removal from aqueous solution by enhanced electrocoagulation process by persulfate salts
Data format	Raw and Analyzed
Type of data	Figure
Data collection	Extraction of data from the electrocoagulation process at the laboratory scale. To maximize the efficiency of fluoride removal, the electrocoagulation process was operated under different operational conditions including solution pH, reaction time, supporting electrolyte concentration, current intensities, initial concentration of fluoride, and dose of persulfate salts.
Data source location	Institution: Saveh University of Medical Sciences City/Town/Region: Saveh, Markazi Province Country: Iran
Data accessibility	Repository name: Mendeley data Data identification number: 10.17632/chmgmkrxwk.1 Direct URL to data: https://data.mendeley.com/datasets/chmgmkrxwk/1

## Value of the Data

• Fluorine (F^–^) is one of the widely distributed elements on Earth and the high F^–^ concentrations in various areas of the world have caused many concerns such as skeletal damage, dental fluorosis, mental disorders in children, osteosclerosis and structural changes in DNA [1,2]. But the F^–^ ion has a unique property, and within permissible limits is beneficial for the production and maintenance of healthy bones and teeth [2]. The contact of surface and subsurface water with mineral sediments and industrial effluent consisting of F^–^ can pollute these water bodies [1], [2], [3]. For F^–^ ions removal various methods including adsorption [4], [5], [6], chemical precipitation [7,8], ion exchange [9], reverse osmosis and nanofiltration [1,10], and electrocoagulation process [11] were previously employed. In the field of water treatment, coagulation and flocculation are crucial processes that involve the aggregation of suspended and colloidal particles into flocs that can rapidly settle to the bottom of a clarifier, leaving a clear supernatant suitable for filtration. [12,13]. The use of chemical coagulants has been found to leave trace amounts of chemicals in the water filtrate, which can lead to long-term ingestion of these chemicals and have been associated with neurodegenerative diseases. This contradicts the purpose of water treatment, making the need for an alternative to these chemicals imperative [12]. To overcome the disadvantages of mentioned methods, the combined process was proposed to achieve high performance and low operational problems [14,15]. The present project was designed on the laboratory scale aiming to optimize fluoride removal by a combination of electrocoagulation with oxidation processes.
• These datasets improve our understanding of the fluoride removal from the aqueous solution by enhanced electrocoagulation process by persulfate salts. Additionally, the effect of different operational conditions including solution pH, reaction time, supporting electrolytes concentration, the intensity of the electric current, initial concentration of fluoride, and dose of persulfate salts on the performance of the electrocoagulation process was systematically investigated.
• Both industrial practitioners and the public community may benefit from this dataset.
• Researchers in environmental science and chemistry can also benefit from these data for testing if coagulation and oxidation processes apply to fluoride removal.

## 1 Data Description

The dataset on fluoride removal from aqueous solution by enhanced electrocoagulation process by persulfate salts is uploaded on Mendeley Data [16]. The dataset is provided as an Excel file including five sheets.

**Fig. 1:**
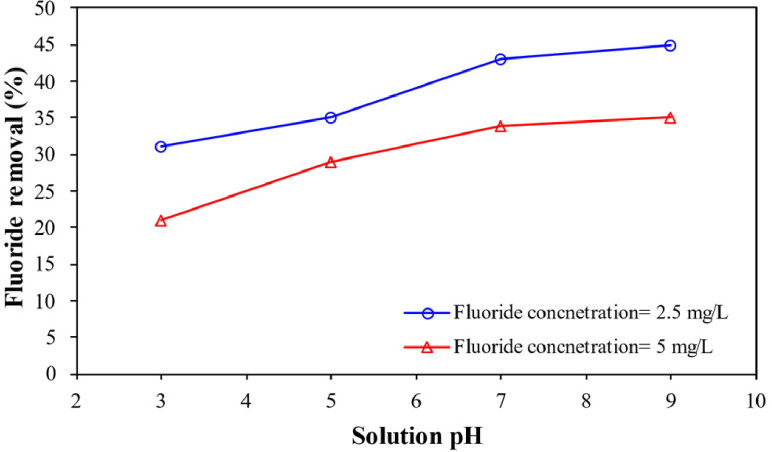
Fluoride removal as a function of solution pH.

**Effect of solution pH** provides information about the fluoride removal by enhanced electrocoagulation process by persulfate salts at various solution pH ranging from 3 to 9 under the experimental condition of electric current= 0.45 A, electrolyte concentration= 1.5 g/L, persulfate dose= 1 mg/L, and 15 min reaction time (Fig. 1).

**Fig. 2:**
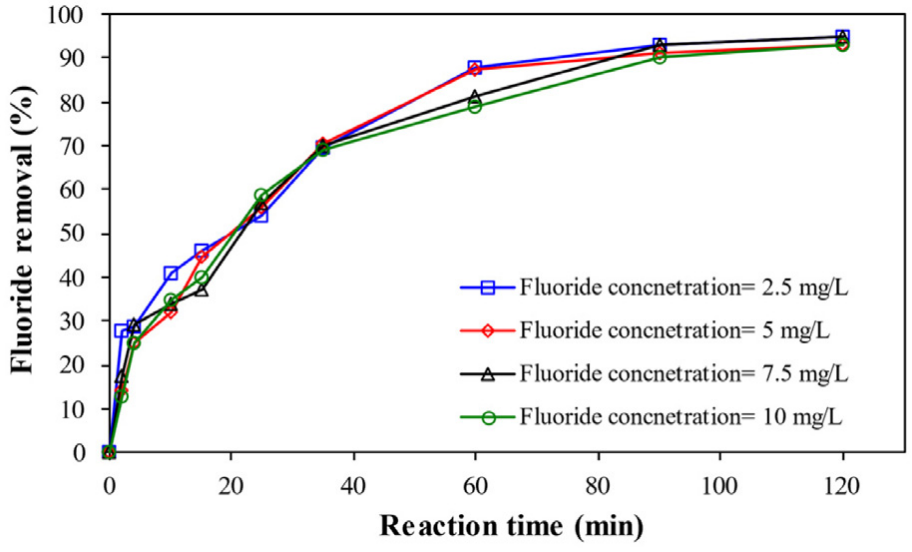
Variation of fluoride removal by progressing the reaction time.

**Effect of reaction time** represents (Fig. 2) the fluoride removal by enhanced electrocoagulation process by persulfate salts by progressing the reaction time under the experimental condition of pH= 7, electric current= 0.45 A, electrolyte concentration= 1.5 g/L, and persulfate dose= 1 mg/L.

**Fig. 3:**
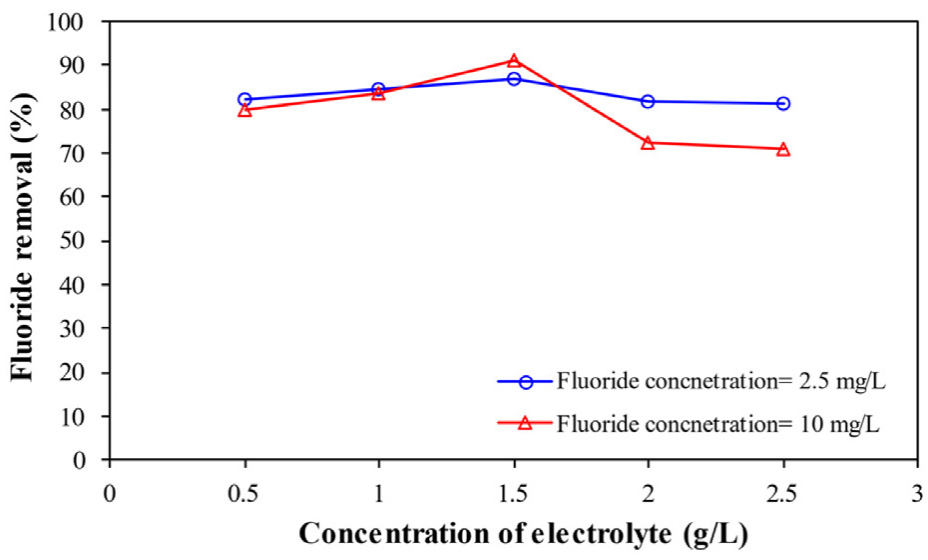
Effect of concentration of electrolyte on fluoride removal.

**Effect of concentration of supporting electrolyte** shows the variation of the performance of enhanced electrocoagulation process by persulfate salts by changing the electrolyte concentrations at pH= 7, electric current= 0.45 A, persulfate dose= 1 mg/L, and 90 min reaction time (Fig. 3).

**Fig. 4:**
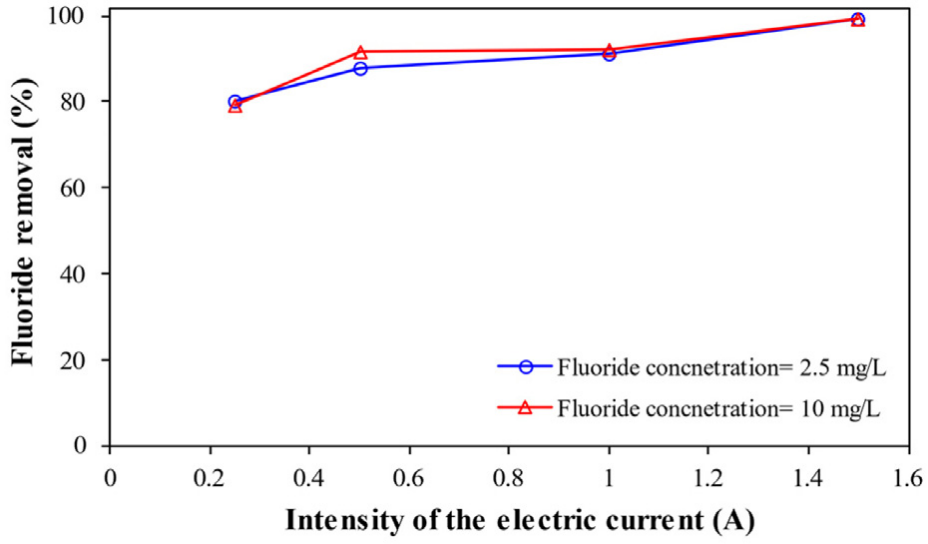
Effect of intensity of the electric current on fluoride removal.

**Effect of the intensity of the electric current** provides information about the variation of fluoride removal of enhanced electrocoagulation process by persulfate salts by changing the intensity of the electric current at pH= 7, electrolyte concentration= 2 g/L, persulfate dose= 1 mg/L, and 90 min reaction time (Fig. 4).

**Fig. 5:**
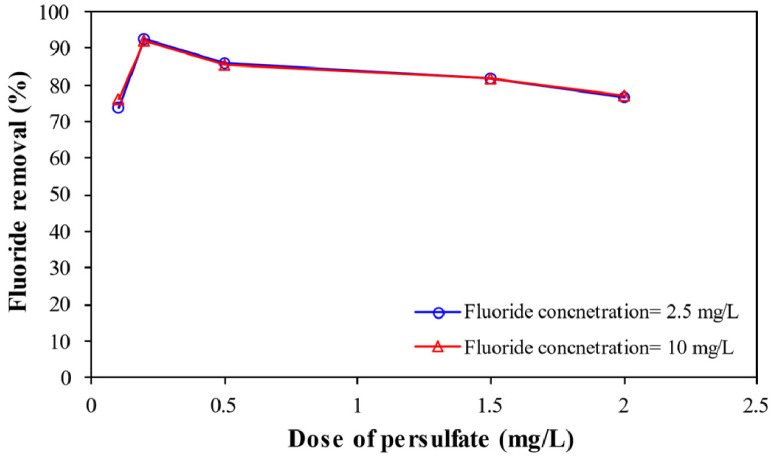
Effect of persulfate dose on fluoride removal.

**Effect of persulfate dose** represents (Fig. 5) the fluoride removal by enhanced electrocoagulation process by persulfate salts by increasing the dose of persulfate under the experimental condition of pH= 7, electric current= 1.55 A, electrolyte concentration= 1.5 g/L, and 90 min reaction time.

## 2 Experimental Design, Materials and Methods

### 2.1 Samples and Materials

The influent samples in the electrocoagulation setup were prepared synthetically. To prepare the stock solution of fluoride, 0.1 g of sodium fluoride salts (CAS #: 7681-49-4, molar mass: 41.99 g/mol, and chemical formula: NaF) were dissolved in 1 L of deionized water (DW). The working solutions were prepared by diluting the stock solution with DW on a daily basis. Sodium peroxy-disulfate (CAS #: 7775-27-1, molar mass: 238.11 g/mol, and chemical formula: Na_2_S_2_O_8_) and sodium chloride (CAS #: 7647-14-5, molar mass: 58.44 g/mol, and chemical formula: NaCl) were used as the main source of sulfate radicals and supporting electrolyte, respectively. The pH of the solution was adjusted using 0.1 N solutions of NaOH and HCl and measured with a digital pH meter (CG 825, Schott, Germany). All chemicals used in the study were of analytical grade and were obtained from Merck Co. (Darmstadt, Germany).

### 2.2 Electrocoagulation Setup

**Fig. 6:**
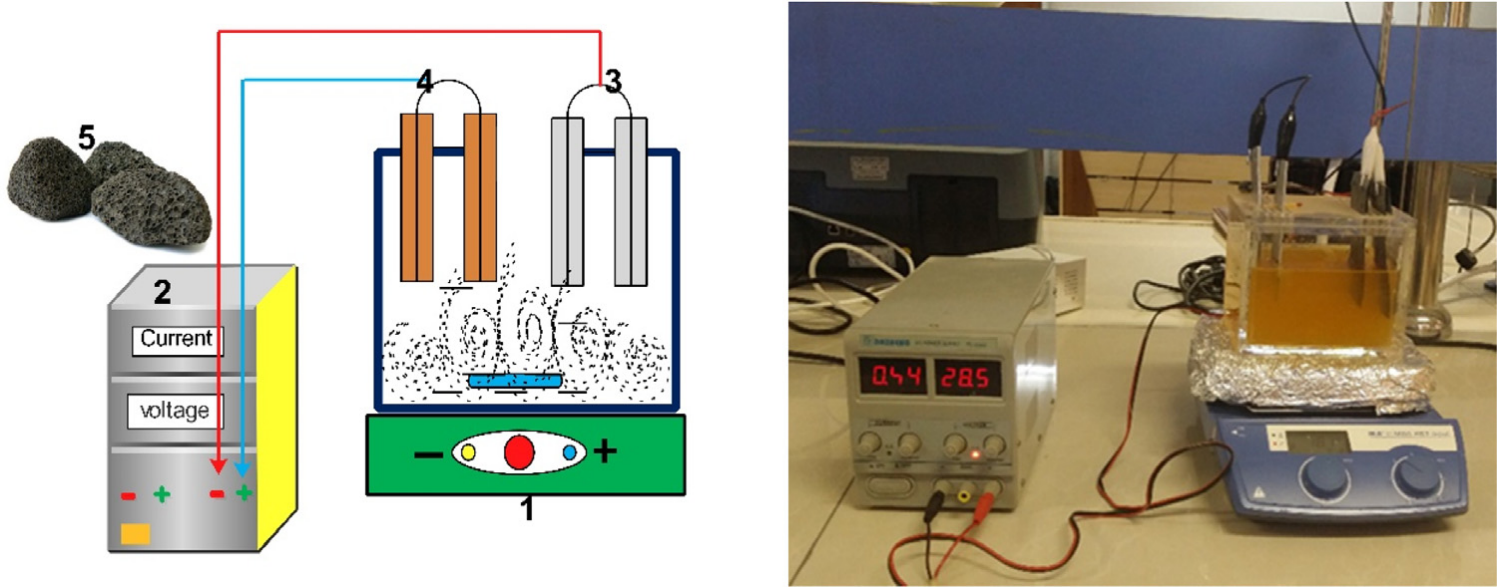
Electrocoagulation setup: (1) magnetic stirrer, (2) power supply, (3) Aluminum electrodes, (4) Iron electrodes, and (5) Persulfate salts

Fig. 6 illustrates both the schematic diagram and the actual setup of the laboratory scale of the electrocoagulation (EC) process. The setup utilized in the experiments consisted of a plexiglass reactor with a total volume of 1000 mL and two pairs of parallel iron and aluminum electrodes (10 cm × 4 cm × 0.3 cm). For typical experiments, 500 mL of the synthetic sample containing F^–^ was transferred into the reactor, and a prespecified concentration of supporting electrolyte and sulfate salts was added. By connecting the electrodes to the power supply (PS-305D, DAZHENG, China) and regulating the intensity of the electric current, the reaction was started. After desire intervals, the suspension was sampled and the residual concentration of F^–^ was determined. To ensure complete mixing, a magnetic stirrer (C MAG HS7, IKA, Germany) was utilized at a speed of 200 rpm during all experiments.

### 2.3 Instrumentation

The F^–^ concentration in the solutions was determined by the 4500- F^–^-D. SPADNS method according to the literature [17] with UV-Visible spectrophotometer (DR6000 Benchtop, HACH, Germany). At first, 10.0 mL of the sample was poured into a dry square cell and pipet 2.0 mL of SPADNS reagent was into a cell and swirled to mix. After 1 min reaction period, the concentration of F^–^ was recorded at 570 nm.

## Limitations

Not applicable.

## Ethics Statement

The data described here did not involve human subjects, animal experiments, or data collection through social media platforms.

## CRediT authorship contribution statement

**Hamid Reza Tashauoei:** Data curation, Visualization, Writing – original draft. **Mokhtar Mahdavi:** Investigation, Data curation, Writing – original draft. **Ali Fatehizadeh:** Conceptualization, Methodology, Writing – review & editing. **Ensiyeh Taheri:** Data curation, Validation, Writing – review & editing.

## Data Availability

Comprehensive dataset on fluoride removal from aqueous solution by enhanced electrocoagulation process by persulfate salts (Original data) (Mendeley Data)

## References

[1] Fatehizadeh A., Amin M.M., Sillanpää M., Hatami N., Taheri E., Baghaei N., Mahajan S. (2020). Modeling of fluoride rejection from aqueous solution by nanofiltration process: Single and binary solution. Desalin. Water Treat..

[2] Malakootian M., Fatehizadeh A., Yousefi N., Ahmadian M., Moosazadeh M. (2011). Fluoride removal using regenerated spent bleaching earth (RSBE) from groundwater: Case study on Kuhbonan water. Desalination.

[3] Sanaei D., Dehghani M.H., Sharifan H., Jain M., Roshan B., Arcibar-Orozco J.A., Inglezakis V.J. (2023). Synthesis of a novel perovskite-carbon aerogel hybrid adsorbent with multiple metal-Lewis active sites for the removal of dyes from water: experimental and DFT studies. New J. Chem..

[4] Mahdavi M., Ebrahimi A., Mahvi A.H., Fatehizadeh A., Karakani F., Azarpira H. (2018). Experimental data for aluminum removal from aqueous solution by raw and iron-modified granular activated carbon. Data Brief.

[5] Taheri E., Fatehizadeh A., Lima E.C., Rezakazemi M. (2022). High surface area acid-treated biochar from pomegranate husk for 2,4-dichlorophenol adsorption from aqueous solution. Chemosphere.

[6] Ahmadian M., Yosefi N., Toolabi A., Khanjani N., Rahimi S., Fatehizadeh A. (2012). Adsorption of direct yellow 9 and acid orange 7 from aqueous solutions by modified pumice. Asian J. Chem..

[7] Crittenden C., Turssel R., Hand D., Howe K., Tchobanoglous G. (2005).

[8] Aoudj S., Khelifa A., Drouiche N. (2017). Removal of fluoride, SDS, ammonia and turbidity from semiconductor wastewater by combined electrocoagulation–electroflotation. Chemosphere.

[9] Zhao Y., Li X., Liu L., Chen F. (2008). Fluoride removal by Fe(III)-loaded ligand exchange cotton cellulose adsorbent from drinking water. Carbohydr. Polym..

[10] Lim H.R., Choo C.M., Chong C.H., Wong V.L. (2021). Optimization studies for water defluoridation with two-stage coagulation processes using new industrial-based chemical coagulants. J. Water Process. Eng..

[11] Hu C.Y., Lo S.L., Kuan W.H., Lee Y.D. (2005). Removal of fluoride from semiconductor wastewater by electrocoagulation–flotation. Water Res..

[12] Tijjani Usman I.M., Ho Y.C., Baloo L., Lam M.K., Show P.L., Sujarwo W. (2023). Comprehensive Review of Modification, Optimisation, and Characterisation Methods Applied to Plant-Based Natural Coagulants (PBNCs) for Water and Wastewater Treatment. Sustainability.

[13] Cheng S.Y., Show P.L., Juan J.C., Chang J.S., Lau B.F., Lai S.H., Ng E.P., Yian H.C., Ling T.C. (2021). Landfill leachate wastewater treatment to facilitate resource recovery by a coagulation-flocculation process via hydrogen bond. Chemosphere.

[14] Hadi S., Taheri E., Amin M.M., Fatehizadeh A., Aminabhavi T.M. (2021). Advanced oxidation of 4-chlorophenol via combined pulsed light and sulfate radicals methods: Effect of co-existing anions. J. Environ. Manage..

[15] Rafiei N., Fatehizadeh A., Amin M.M., Pourzamani H.R., Ebrahimi A., Taheri E., Aminabhavi T.M. (2021). Application of UV/chlorine processes for the DR83:1 degradation from wastewater: effect of coexisting anions. J. Environ. Manag..

[16] Fatehizadeh A., Tashauoei H.R., Mahdavi M., Taheri E. (2023).

[17] Rice E.W., Bridgewater L. (2012).

